# Association of Zika Virus with Myocarditis, Heart Failure, and Arrhythmias: A Literature Review

**DOI:** 10.7759/cureus.1399

**Published:** 2017-06-27

**Authors:** Abdul M Minhas, Asra Nayab, Shobana Iyer, Mehek Narmeen, Kaneez Fatima, Muhammad S Khan, Jonathan Constantin

**Affiliations:** 1 Internal Medicine, Orange Park Medical Center; 2 Radiology, Orange Park Medical Center; 3 Department of Internal Medicine, Dow University of Health Sciences, Karachi, Pakistan; 4 Internal Medicine, John H Stroger J. Hospital of Cook County; 5 Cardiology, Orange Park Medical Center

**Keywords:** cardiovascular complications, zika virus

## Abstract

As the concerns regarding Zika virus (ZIKV) are mostly of neurological disorders, especially in neonates and infants, other possible threats of the virus may have been overlooked. Our study focuses on the potential threat that ZIKV may pose to the heart like that of similar arboviral diseases. We conducted a literature search of multiple terms in March 2017 using the search engines, PubMed, Embase, and SCOPUS. Articles were then reviewed by two independent reviewers, adhering strictly to our review criteria. No discriminations were made whether the studies were conducted on human or non-human subjects. Three relevant studies were shortlisted and finalized. The nature of the studies is as follows: prospective observational multicenter study (n = 9), case report (n = 1), and animal studies (n = 5). The studies suggested an association between cardiovascular complications and ZIKV in the acute phase of the infection. We recognize the limitations of our study owing to the paucity of the sample size of our literature review. However, the significant findings have also proved the dire need to conduct more research to further back the possibility of ZIKV’s influence on the cardiac cells. Closely studying these associations can eventually help develop a vaccine for ZIKV in the near future.

## Introduction and background

The Zika virus (ZIKV) has reportedly existed in humans since 1954 [1], but it was not until February 2016 that the World Health Organization (WHO) declared it a public health emergency of international concern (PHEIC) [2]. This apprehension came about with an increase in the number of cases, mainly pertaining to the neurological complications [3]. To better understand ZIKV [4], the scientific community then began to extensively study the virus [5], its mode of transmission [6-7], and any other implications it may have on living organisms [8]. Amongst many things [9-12], the studies also led to the discovery that the diameter of the ZIKV was 40 nm [13], smaller than that of the dengue virus [14]. Since it has already been established that similar arboviral diseases, such as chikungunya and dengue, have acute myocardial implications [15], it was suggested that due to the smaller size of the ZIKV, the virus also has the ability to invade cardiac cells [16]. Owing to the recent state of emergency that the virus has created [17] and the lack of time lapse since then [18], the effects that ZIKV may have on cardiac cells have been overlooked [19]. Our study aims to draw attention towards this untapped, yet clinically important feature of ZIKV.

## Review

Methodology

An extensive systemic literature search was conducted in March 2017 using search engines (PubMed, Embase, and SCOPUS) to find literature on the terms, Zika virus AND myocarditis OR arrhythmias OR  cardiovascular OR heart failure. All existing data from the inception of each database until the given month and year was extracted. Any article that included the search terms in the title and/or abstract, were included for further reviewing. Practical guidelines, randomized controlled trials, clinical trials, meta-analyses, systematic reviews, presentations, observational studies, and case reports were part of our inclusion criteria. No restrictions were put on whether the subjects of the study were humans or non-humans. However, articles that were letters to the editor, editorials, were written in a language other than English, or not accessible on digital media were excluded.

Each article was evaluated by two independent reviewers who were blinded to the findings of each other. The assessments were made on the formerly stated eligibility criteria. Full texts of articles that were relevant were extracted for further evaluation. A consensus through discussion was employed if discrepancies regarding the eligibility of a study occurred. The summaries of our findings are presented in Table 1.

**Table 1:** Summary of the Three Studies cMRI: cardiac magnetic resonance imaging; CPK: creatine phosphokinase; ECG: electrocardiogram; H&E: hemotoxylin and eosin; MAC ELISA: IgM antibody capture enzyme-linked immunosorbent assay; MRI: magnetic resonance imaging; PRNT: plaque reduction neutralization test; rRT-PCR: real-time reverse transcriptase-polymerase chain reaction

Study	Study Type	First Author	Country of Corresponding Author (Year of Publication)	Number of specimen	Human/Non-human Specimen	Virus Inoculated	Relevant Tests Done
1	Case Report	Aletti M [19]	France (2016)	1	Human	No	Troponin I, CPK level, echocardiogram, ECG, MRI, MAC-ELISA, indirect ELISA
2	Prospective Observational Multicenter Study	Carta KAG	Venezuela (2017)	9	Human	No	ECG, laboratory (including virological) studies, echocardiogram, cMRI, Holter monitoring
3	Animal Study	Xiao-Feng Li [20]	China (2016)	5	Non-human	Yes	ELISA, H&E staining, rRT-PCR, PRNT

Results

Our literature search revealed only three relevant studies; a prospective observational multicenter study (Carta KA, Mendoza I, Morr I, Misticchio F, Meza Y, Finizola V, et al.: Myocarditis, Heart Failure and Arrhythmias in Patients with Zika. Presented at the American College of Cardiology's 66th Annual Scientific Session, Washington, DC, 2017 and available from http://www.sciencedirect.com/science/article/pii/S073510971734295X), a case report [19], and an animal study [20]. The number of cases exposed to the virus added up to 15; nine humans (six females, three males), one human (male), and five rhesus monkeys (sex unidentified), respectively.

Study 1

A 45-year-old healthy male with a recent history of traveling to the French West Indies arrived at an outpatient department (OPD) in France with complaints of fever, diffuse joint pain, myalgia, headache, and diarrhea over the past five days [19]. He also complained of a mild (scoring 4/10 on the pain assessment tool) throbbing, squeezing mediothoracic localized pain and no dyspnea. On examination, it was revealed that the body temperature and heart rate were above normal. The presence of bilateral conjunctivitis and hand edema were noted, but there was an absence of skin rash, signs of cardiac failure, or abnormal heart sounds. Blood samples came back positive for ZIKV.

Myocarditis was diagnosed owing to an increased level of troponin I and creatine phosphokinase (CPK), as well ST- segment elevation in the anteroseptal region. However, treatment with bisoprolol and ramipril quickly normalized the elevated levels of the enzymes, as well as diminished chest pain and electrocardiographic changes. Paracetamol resolved his fever and conjunctivitis three days after the start of symptomatic treatment. A cardiac magnetic resonance imagining (cMRI) taken 10 days later showed a slight left ventricular dilatation with no other abnormalities.

Study 2

This prospective observational multicenter study with a six-month follow-up looked into the possibility of cardiovascular (CV) complications of nine adult cases (six females, three males) in Venezuela. All cases were positive for ZIKV (using virological studies), had a mean age of 47 ± 17 years, and had no previous cardiac history (only one had controlled hypertension). With the help of an electrocardiogram (ECG), echocardiogram, Holter monitoring, and cMRI, it was established that eight of the cases had arrhythmias and six presented with heart failure. Of the eight arrhythmias, three were acute atrial fibrillation (two paroxysmal, one persistent), two were non-sustained atrial tachycardia, and two were ventricular arrhythmias. Five of the six heart failure patients had a low ejection fraction (EF), and one had preserved EF with pre-eclampsia and moderate to severe pericardial effusion.

Study 3

Following the protocols and biosafety of the governing/local bodies in China, a ZIKV strain was isolated from a human and subcutaneously (s.c.) inoculated into five 5-year-old healthy rhesus monkeys [20]. In the 34-day long observational period, two s.c. inoculated rhesus monkeys were euthanized at days 5 and 10 postinfection (p.i.), for immunohistological and pathological purposes. Body temperature, blood cell count, and blood chemistry, as well as excretion of viral RNAs in the blood, urine, saliva, and lacrimal fluid, were recorded at regular intervals. The necropsies on day 5 revealed the presence of viral RNA in the central nervous system and the visceral organs, inclusive of the heart. The positive, as well as negative strands of viral RNA, were also present in the heart. However, on day 10 p.i., it was revealed that other than the spinal cord, spleen, lymph node, liver, kidney, pancreas, and stomach, all other organs were clear of viral RNA.

Discussion

Zika virus infections occasionally induce certain neurotropic disorders (e.g., Guillain-Barre syndrome [11], acute myelitis [21], meningoencephalitis [22], and neurological complications, such as microcephaly) [23]. In the acute phase of the infection, the virus also induces symptoms similar to other flaviviruses, such as the dengue virus, West Nile virus, yellow fever virus, Japanese encephalitis virus, and tick-borne encephalitis virus [24]. The range of symptoms and complications suggest the diversity and the widespread effect it has on living organisms and the need to further evaluate the extent of its harm.

The 15 cases in our study suggest that, apart from the classical clinical presentations of maculopapular skin rash, headaches, low-grade fever, arthralgias, myalgia, and conjunctivitis [25], ZIKV may also have symptoms of a CV nature. This can prove to be fruitful in cases where ZIKV may be asymptomatic but the patient may have a history of traveling to ZIKV-prone areas. Increased cardiac enzyme levels may help indicate the presence of the virus, and the patient can be put in isolation to avoid the spread as a precautionary measure [26].

ZIKV is infamously known to cause fetal neurological complications, namely microcephaly, where the mother is exposed to the virus during the gestational period [27-29]. Interestingly, Brasil, et al. suggested in their study the possibility of an association between congenital heart disease in an infant born to a ZIKV-infected mother [30]. Furthermore, our study also suggests that ZIKV may also pose a threat to the heart of the mother, adding burden to the existing physiological changes of the organ during her pregnancy [31]. This is of paramount importance since CV complications are the most common cause of mortality in pregnancy [32]. Considering the weight of the two suggested the hypotheses that there is a dire need for more evidence-based research.

Knowing that the virus has an influence on the heart and does not have a sudden onset, careful monitoring of its function and its prevention can reduce the rate of mortality.

Lastly, the animal study conducted by Li, et al. plays a pivotal role in providing a draft for developing vaccines for ZIKV in the near future. The non-human primates give an immune response to flavivirus infections closest to that of humans [33]. Preventing an infection from ZIKV can go a long way to reduce the burden on healthcare systems and improve the quality of life.

Our study is subjected to limitations that need to be addressed. Firstly, the study does not represent a large population as the sample size is incredibly small. Second, certain articles that may have addressed the topic at hand but whose extracts were not accessible were not included for evaluation.

## Conclusions

Of the limited pool of studies available in the field of cardiology in association with ZIKV, we can see a direct link between the two. However, there is not enough evidence to draw a conclusive hypothesis, owing to the scarce clinical research and data available. Our study aims to encourage fellow clinicians and researchers to further investigate into the prospects of CV complications with respect to ZIKV. In-depth research may also eventually lead the scientific community into finding a vaccine for ZIKV.
